# Neonatal Kaposiform Hemangioendothelioma with Kasabach–Merritt Phenomenon Presenting as Severe Airway Obstruction at Birth: A Case Report

**DOI:** 10.3390/children12111429

**Published:** 2025-10-23

**Authors:** Soyoung Shin, Ye Jee Shim

**Affiliations:** 1 Department of Pediatrics, Keimyung University School of Medicine, Daegu 42601, Republic of Korea; 2 Department of Pediatrics, School of Medicine, Kyungpook National University, Daegu 41944, Republic of Korea

**Keywords:** kaposiform hemangioendothelioma, Kasabach–Merritt phenomenon, neonatal airway obstruction, sirolimus, vascular tumor

## Abstract

**Highlights:**

**What are the main findings?**

- Kaposiform hemangioendothelioma with Kasabach–Merritt phenomenon can cause severe neonatal airway obstruction at birth.
- Sirolimus therapy led to rapid hematologic recovery and significant tumor regression in the acute neonatal period.

**What is the implication of the main finding?**

- Prompt airway management and early recognition of consumptive coagulopathy are crucial for survival.
- Sirolimus-based therapy is an effective treatment option for neonatal KHE complicated by Kasabach–Merritt phenomenon.

**Abstract:**

**Background/Objectives**: Kaposiform hemangioendothelioma (KHE) is a rare, locally aggressive vascular tumor of infancy, often complicated by Kasabach–Merritt phenomenon (KMP), a consumptive coagulopathy characterized by severe thrombocytopenia and hypofibrinogenemia. Airway involvement at birth is exceptionally rare and can be life-threatening. This study reports the clinical presentation and treatment course of a full-term male neonate with severe airway obstruction caused by KHE with KMP. **Case Presentation**: The patient had unremarkable prenatal imaging but presented at birth with severe respiratory distress requiring emergent intubation. Physical examination revealed firm violaceous swelling over the right cervicothoracic region. Laboratory tests showed profound thrombocytopenia (22,000/μL), hypofibrinogenemia (75 mg/dL), and coagulopathy. Imaging findings were consistent with KHE complicated by KMP. Due to bleeding risk, the biopsy was not performed. Initial treatment included platelet and plasma transfusions, intravenous immunoglobulin (IVIG), corticosteroids, and antithrombin III replacement. Vincristine was discontinued owing to gastrointestinal toxicity. Sirolimus therapy was initiated on day 14. Following sirolimus initiation, rapid platelet recovery was observed. At three months, marked tumor regression was documented. After mild recurrence, sirolimus was reintroduced, and the patient remained stable at 16-month follow-up. **Conclusions**: This case underscores the critical importance of prompt airway stabilization, early recognition of consumptive coagulopathy, and sirolimus-based therapy in managing neonatal KHE with airway involvement.

## 1. Introduction

Kaposiform hemangioendothelioma (KHE) is a rare, locally aggressive vascular tumor of infancy and childhood, characterized histologically by spindle-shaped endothelial cells and clinically by an infiltrative growth pattern without spontaneous regression [1,2]. The tumor has a strong predilection for the skin, deep soft tissues, and retroperitoneum, and may involve critical anatomical structures depending on its location. Approximately 70% of KHE cases are complicated by Kasabach–Merritt phenomenon (KMP), a consumptive coagulopathy caused by intralesional platelet trapping and activation, leading to profound thrombocytopenia, hypofibrinogenemia, and life-threatening bleeding [3,4].

While cutaneous or musculoskeletal involvement is most common, head and neck localization—particularly when involving the airway—is exceedingly rare. Neonatal airway compromise due to KHE is a medical emergency, requiring immediate airway stabilization, rapid recognition of KMP, and prompt initiation of systemic therapy [5,6,7]. The optimal therapeutic approach remains challenging, with vincristine, corticosteroids, and sirolimus representing the most widely used pharmacologic agents [8,9,10].

Here, we present a term male neonate with cervicothoracic KHE complicated by severe airway obstruction at birth and KMP, successfully managed with early sirolimus initiation after limited steroid and vincristine response. This case underscores the importance of multidisciplinary care, rapid diagnosis, and individualized treatment in managing this rare but life-threatening presentation.

## 2. Case Presentation

**Figure 1:**
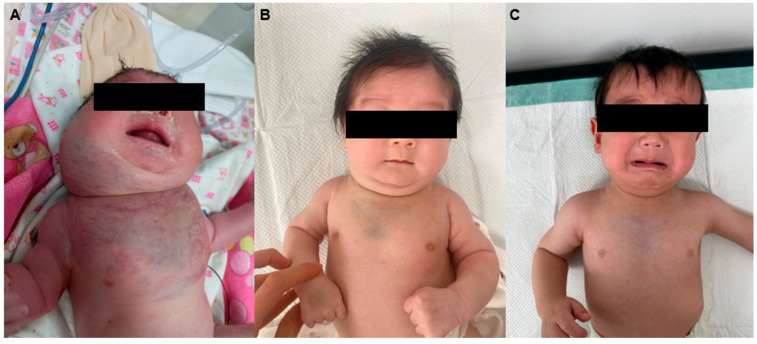
Clinical photographs of the patient. (**A**) Immediately after birth, showing a large cervical mass causing severe airway obstruction. (**B**) At 2 months of age, after NICU discharge, demonstrating partial regression of the mass. (**C**) At 1 year of age, showing significant regression with minimal residual swelling.

A term male neonate was born via spontaneous vaginal delivery following an uncomplicated pregnancy. The mother’s prenatal course was generally uneventful, with no structural or vascular abnormalities on serial ultrasounds. Mild scalp edema and polyhydramnios led to her transfer at 32 weeks, but no vascular malformations were detected after that, and prenatal ultrasound images of airway or chest vascular anomalies are unavailable due to a lack of detailed scans. However, immediately after birth, the infant exhibited signs of severe respiratory distress, including prominent inspiratory stridor, cyanosis, and episodes of apnea. Emergency endotracheal intubation was performed in the delivery room to secure the airway, after which the patient was transferred to the neonatal intensive care unit (NICU) for stabilization and further evaluation. At this time, marked swelling with violaceous discoloration and edema was noted over the right cheek, cervical region, and anterior chest wall (Figure 1A).

On admission, physical examination revealed firm, non-pulsatile, and infiltrative swelling extending into the supraclavicular fossa and upper thoracic region. Initial laboratory evaluation demonstrated profound thrombocytopenia, with a platelet count of 22,000/μL, and severe hypofibrinogenemia at 75 mg/dL. Coagulation studies revealed prolongation of both the prothrombin time and activated partial thromboplastin time, findings consistent with disseminated intravascular coagulation secondary to consumptive coagulopathy.

**Figure 2:**
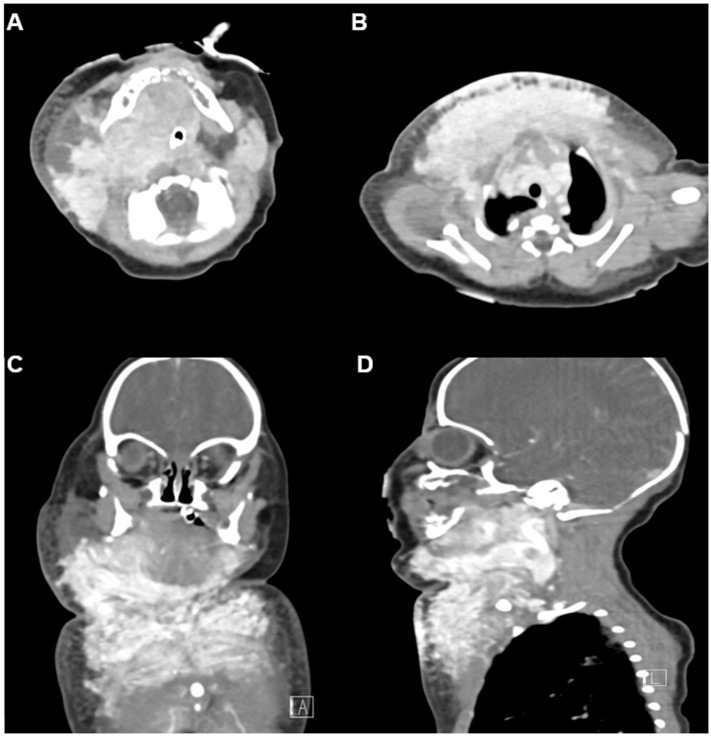
Initial contrast-enhanced CT at 5 days of age. (**A**,**B**) Axial and mediastinal views demonstrate an intensely enhancing infiltrative lesion involving the right parotid, submandibular, lingual, retropharyngeal, posterior cervical, and both anterior deep soft tissue layers, extending into the upper anterior chest wall. Numerous feeding and draining superficial and deep vessels are evident, consistent with a vascular tumor. Endotracheal tube is in situ. (**C**) Coronal and (**D**) sagittal views further delineate the mass with associated subsegmental atelectasis in the left lower lobe.

Neck and chest ultrasonography demonstrated a diffuse, infiltrative, and hypervascular soft tissue mass without cystic components. Contrast-enhanced CT performed at 5 days of life revealed an intensely enhancing infiltrative lesion involving the right parotid, submandibular, lingual, and retropharyngeal spaces, with extension into the posterior cervical compartments and both anterior deep soft tissue layers, further involving the upper anterior chest wall. The respiratory distress was considered to be due to external compression by the lesion rather than direct invasion of the upper airway. Numerous feeding and draining vessels were visualized (Figure 2A–D). These findings, together with the hematologic profile and clinical presentation, strongly supported a diagnosis of kaposiform hemangioendothelioma (KHE) complicated by Kasabach–Merritt phenomenon (KMP). Maternal-fetal antibody screening and immunologic workup were unremarkable, effectively excluding alloimmune thrombocytopenia and other hematologic disorders. Given the severe coagulopathy and associated high risk of hemorrhage, surgical biopsy was deemed unsafe, and a clinico-radiographic diagnosis was established.

Endotracheal intubation was performed immediately after birth due to respiratory distress caused by airway obstruction. The patient was managed with synchronized intermittent mandatory ventilation (SIMV) for 14 days. Successful extubation was achieved on day 14, followed by one day of noninvasive nasal continuous positive airway pressure (CPAP), after which respiratory support was discontinued. Neck CT findings indicated the airway obstruction resulted predominantly from extrinsic compression by the infiltrating hemangioma. However, because of severe thrombocytopenia, invasive bronchoscopy to assess for intraluminal airway infiltration could not be performed, representing a limitation in the diagnostic workup.

The patient’s acute management focused on correcting the coagulopathy and supporting vital functions. Daily transfusions of platelet concentrates and fresh frozen plasma were administered to maintain hemostatic parameters. Additional measures included intravenous immunoglobulin, vitamin K supplementation, high-dose methylprednisolone, and antithrombin III replacement, all aimed at controlling the disseminated intravascular coagulation and consumptive coagulopathy. Mechanical ventilation was maintained in synchronized intermittent mandatory ventilation (SIMV) mode to ensure adequate oxygenation. Nutritional support was initiated via enteral feeding, and continuous intravenous fentanyl infusion was employed for sedation and analgesia.

High-dose methylprednisolone therapy was commenced on the first day of life. Vincristine was introduced early in the course; however, it was discontinued shortly thereafter due to gastrointestinal toxicity. Because of the persistence of coagulopathy and signs of tumor progression, sirolimus therapy was initiated on day 14, with dosing titrated according to published consensus guidelines. Remarkably, by the same day, platelet counts had improved to above 200,000/μL and remained stable thereafter on sirolimus monotherapy.

**Figure 3:**
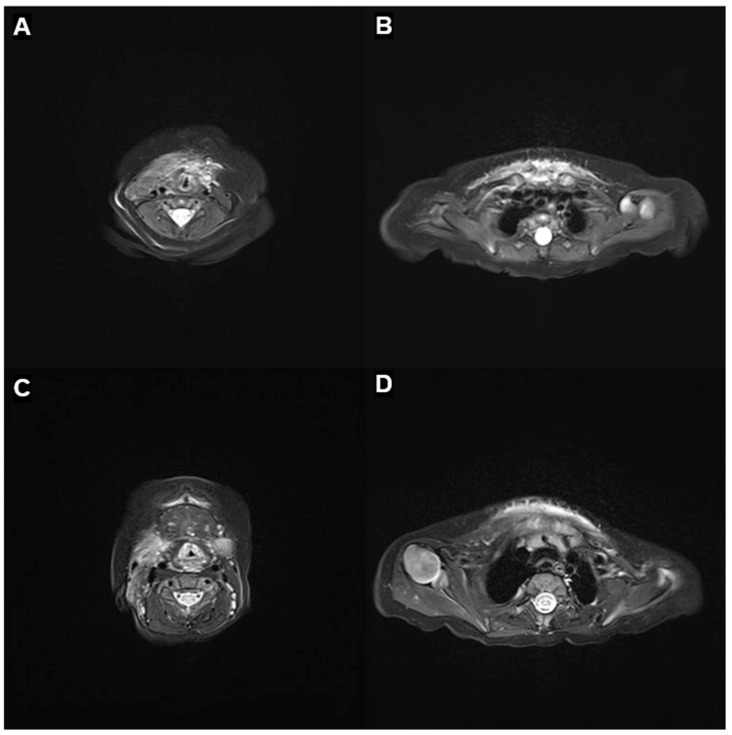
Follow-up MRI after sirolimus therapy. (**A**,**B**) At 3 months of age, axial T2-weighted neck and mediastinal images show marked regression of the previously infiltrative hypervascular lesion, with residual involvement from the infrahyoid to the distal sternum. The great vessels are preserved in their normal positions. Several reactive lymph nodes are noted in the right level V, and subsegmental atelectasis persists in the left lower lobe. (**C**,**D**) At 9 months of age, axial T2-weighted neck and mediastinal images demonstrate further regression of the vascular tumor, including resolution of the right parotid involvement. A subtle residual lesion persists in the lower neck and upper mediastinum encircling the thoracic inlet, but without airway compression.

At 2 months of age, following clinical improvement and resolution of the acute phase, the patient was discharged from the NICU (Figure 1B). MRI of the neck and mediastinum performed at 3 months revealed a marked reduction in the hypervascular infiltrating lesion, extending from the infrahyoid level to the distal sternum, with preservation of the great vessels and no evidence of airway compromise (Figure 3A,B). At 9 months, MRI showed further regression with resolution of right parotid involvement but subtle residual lesions encircling the thoracic inlet in the lower neck and upper mediastinum, without airway obstruction (Figure 3C,D). At 1 year of age, clinical photographs demonstrated significant regression of the swelling with minimal residual deformity (Figure 1C). At the most recent follow-up at 16 months, the lesion remained stable without a transfusion requirement.

Cardiac evaluation with serial echocardiography revealed mild, reversible left ventricular systolic dysfunction during the acute phase, with no evidence of direct tumor infiltration into the myocardium or great vessels. In terms of immunization, the patient received all recommended inactivated vaccines in accordance with the national vaccination schedule, while administration of live-attenuated vaccines was deferred throughout the course of sirolimus therapy, in line with established immunosuppression safety guidelines.

## 3. Discussion

Kaposiform hemangioendothelioma with Kasabach–Merritt phenomenon represents a severe clinical entity with significant morbidity and potential mortality, particularly in the neonatal period. The combined burden of tumor mass effect, coagulopathy, and, in rare cases, airway compromise presents unique challenges for diagnosis and management. Airway involvement by KHE is exceedingly uncommon, and presentation at birth is even rarer, with few cases documented in the literature [5,6,7]. In the present case, the absence of abnormal findings on prenatal imaging underscores the possibility that lesions may be either small or radiologically indistinct in utero, only becoming clinically apparent when postnatal circulatory and respiratory dynamics exacerbate airway compromise.

The diagnosis of KHE with KMP is generally based on the integration of clinical, laboratory, and imaging findings, as tissue biopsy often carries unacceptable hemorrhagic risk in patients with severe thrombocytopenia and hypofibrinogenemia [5,8]. Imaging features, such as a poorly circumscribed, infiltrative, hypervascular soft tissue mass without cystic components, are highly suggestive when combined with laboratory evidence of consumptive coagulopathy. In our patient, these findings, along with exclusion of other causes of neonatal thrombocytopenia, enabled a confident diagnosis without histologic confirmation.

It is necessary to expand the scope of differential diagnosis to include vascular malformations. These anomalies present diverse clinical and radiological features that can overlap with other cervicofacial vascular anomalies, making an accurate diagnosis essential. Recent comprehensive reviews, such as the study referenced, systematically summarize the classification, diagnosis, and management of cervicofacial vascular anomalies, including venous, lymphatic, and arteriovenous malformations [11]. Incorporating this perspective enables a more thorough approach in the evaluation and treatment planning of such lesions.

**Figure 4:**
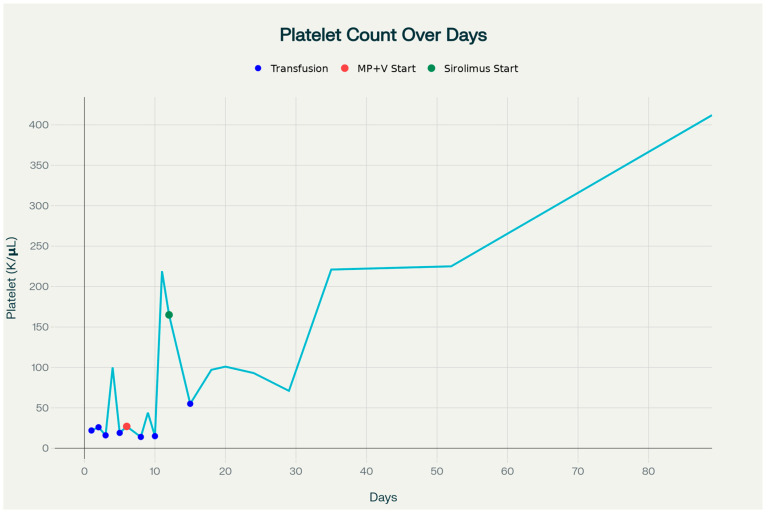
Platelet count trends during the first 89 days of life. Blue dots indicate platelet transfusions. The red dot marks the start of methylprednisolone and vincristine therapy on day 6, and the green dot marks the initiation of sirolimus therapy on day 12. Initial platelet improvement was associated with corticosteroid and vincristine treatment, while sustained recovery occurred following sirolimus administration.

Historically, systemic corticosteroids and vincristine have been the mainstays of treatment for KHE with KMP [9]. However, responses to steroids are often incomplete, and vincristine’s slow onset of action, combined with its potential for neurotoxicity and gastrointestinal side effects, can limit its utility in critically ill neonates [10]. Sirolimus, an oral mTOR inhibitor, has emerged over the past decade as a highly effective alternative, demonstrating rapid hematologic recovery, tumor regression, and an acceptable safety profile in both prospective studies and real-world series [12]. In our patient, as shown in Figure 4, the patient’s platelet counts were 22,000/μL at birth, declining to 14,000/μL by one week of age, followed by an increase to 165,000/μL around day 12, which is likely attributable to methylprednisolone and vincristine therapy. However, the platelet count decreased again around day 15 before gradually rising. With the initiation of sirolimus therapy on day 14, a subsequent normalization of platelet counts and significant tumor reduction were observed within three months. This pattern suggests that the initial platelet improvement was predominantly due to corticosteroid and vincristine treatment, whereas the sustained recovery following three weeks of age can be attributed to the therapeutic effects of sirolimus [13]. The recurrence observed at six months after sirolimus taper is consistent with literature reports indicating that relapse rates can be as high as 40% upon discontinuation, supporting the need for prolonged therapy in select patients [14].

The management of KHE with KMP requires not only tumor-directed therapy but also meticulous supportive care. Transfusion support, correction of coagulation parameters, and careful monitoring for bleeding are essential to stabilize patients before and during systemic treatment. In cases involving the airway, early and secure airway management is paramount, and multidisciplinary coordination among neonatology, pediatric hematology–oncology, otolaryngology, anesthesiology, and radiology teams is vital. Our patient’s favorable outcome—stable disease and transfusion independence at 16 months—was achieved through such an integrated approach, with sirolimus playing a central role in disease control.

Although the prognosis of KHE with KMP has improved markedly in the sirolimus era, long-term follow-up is necessary to monitor for disease recurrence, manage potential drug-related adverse effects, and assess functional outcomes, particularly in patients with lesions involving critical structures such as the airway. This case reinforces that early sirolimus initiation should be considered in neonates with life-threatening KHE, especially when conventional therapies are ineffective or poorly tolerated.

## 4. Conclusions

This case illustrates a rare but critical presentation of neonatal KHE with KMP manifesting as airway obstruction at birth. The successful outcome underscores that prompt airway management, early detection of coagulopathy, and timely initiation of sirolimus can be lifesaving. Multidisciplinary collaboration and long-term follow-up are essential in managing these high-risk patients.

One limitation of this study is that a biopsy could not be performed due to the risk of bleeding, which restricted histopathological confirmation. Additionally, genetic testing was not conducted due to cost constraints, limiting molecular diagnostic insights. These limitations highlight the need for cautious interpretation of the findings and suggest the benefit of future studies incorporating these diagnostic approaches.

## Data Availability

The data presented in this study are available on request from the corresponding author due to patient privacy and institutional ethical restrictions.
